# Evaluation of a Residential Managed Alcohol Program for Aboriginal Peoples Experiencing Homelessness and Alcohol Dependence: Short‐Term Impacts of an Australian Trial

**DOI:** 10.1111/dar.70025

**Published:** 2025-09-02

**Authors:** Kirrilly Thompson, Gianluca Di Censo, Jacqueline Bowden, Neophytos Georgiou, Mark Thompson, Victoria Cock, Blaire Brewerton, Courtney Ryder

**Affiliations:** ^1^ National Centre for Education and Training on Addiction Flinders University Adelaide Australia; ^2^ Flinders University, College of Medicine and Public Health Flinders Health and Medical Research Institute Adelaide Australia; ^3^ School of Medicine and Public Health University of Newcastle Newcastle Australia; ^4^ Drug and Alcohol Services South Australia Adelaide Australia

**Keywords:** Aboriginal and Torres Strait Islander, First Nations, harm reduction, Indigenous, supervised alcohol provision program

## Abstract

**Introduction:**

Homelessness and alcohol dependence can be barriers to accessing essential services such as health care, housing, and social supports. Managed alcohol programs (MAP) have emerged as an effective harm reduction strategy for people experiencing homelessness and alcohol dependence. The aim of the present study was to evaluate the short‐term impacts of the first MAP run in South Australia outside of COVID‐19 restrictions and the first in Australia to be conducted in a healthcare setting. It was designed to be culturally appropriate for Aboriginal and Torres Strait Islander clients.

**Methods:**

Descriptive quantitative analysis and an inductive content analysis of case notes for 21 clients who stayed at least one night in the South Australian MAP.

**Results:**

Clients were mostly Aboriginal, female, of middle age and managing multiple health conditions. The median stay was 15 nights per client. The MAP contributed to client wellbeing broadly across five interconnected areas: culture, housing, medical support, government system navigation, and the building of capacity, resilience, and social connectedness.

**Discussion and Conclusions:**

The South Australian MAP provided various interconnected short‐term benefits relevant to people experiencing homelessness and alcohol dependence in general and Aboriginal peoples additionally experiencing the on‐going impacts of colonisation in particular. This evaluation supports international literature on the value of MAPs as an effective harm reduction approach to co‐occurring homelessness and alcohol dependence and strengthens evidence for the feasibility, acceptability, and benefits of MAPs in Australia.

## 1 Introduction

People experiencing homelessness and alcohol dependence often experience higher levels of injury, chronic disease, hospitalisations, mortality, and criminal system contact [1, 2]. This is exacerbated for Aboriginal and Torres Strait Islander peoples [3, 4], as a consequence of colonisation, marginalisation, and racism [5, 6, 7]. Homelessness and substance use can be barriers to accessing essential services, such as health care [8], housing, and social supports [9, 10]. Managed alcohol programs (MAP) have emerged as an effective harm reduction strategy for people experiencing homelessness and alcohol dependence [9, 11, 12, 13, 14, 15, 16, 17], as they are not contingent upon abstinence. MAPs involve the provision of regulated amounts of alcohol at set times in settings such as shelters, day programs, residential rehabilitation programs, transitional housing, and in‐patient settings. They provide a safe and supervised environment for stabilising alcohol consumption and connecting people with housing, health, and social services [18].

Despite the broad and interconnected health and wellbeing benefits of MAPs [8, 11, 19], they are still considered a relatively novel harm reduction intervention. While a MAP providing transitional accommodation for Aboriginal and Torres Strait Islander clients has been operating in remote Queensland in Australia since 2003 [20], there have been no published evaluations to document specific benefits or acceptability in Australia. The aim of the present study was to evaluate a South Australian trial with a focus on the characteristics of clients, program engagement, and the short‐term benefits of participation. To our knowledge, this is the first evaluation of a MAP in Australia.

## 2 Methods

### 2.1 Program Description

For 12 weeks from August to November 2023, Drug and Alcohol Services of South Australia (DASSA) provided the first voluntary MAP in a healthcare setting outside of a COVID‐19 emergency response [21] for First Nations peoples living in the Adelaide parklands who were experiencing homelessness and long‐term alcohol dependence. Referrals were centralised through Drug and Alcohol Services South Australia's Aboriginal Connection Program team that conducts proactive mobile outreach to Aboriginal and Torres Strait Islander peoples. Admission was voluntary and subject to clients having a blood alcohol content under 0.2, not showing signs of intoxication, and being able to provide informed consent to the unit guidelines, which were provided in native languages. Alcohol was provided according to a protocol designed to lead to a peak blood alcohol content of approximately 0.09–0.1. A modified protocol was provided for clients with impaired liver function and liver disease. This involved a reduction or (less commonly) complete absence of alcohol. Where clinically indicated, withdrawal was overseen by medical staff and support was provided, including medication (e.g., diazepam).

### 2.2 Study Design

A mixed‐methods retrospective analysis was undertaken of de‐identified client notes authored by case managers, cultural navigators,^1^ and substance misuse workers. The evaluation was overseen by an Aboriginal Governance Group (AGG) comprising six members identifying as Aboriginal or Torres Strait Islander and chaired by CR.

### 2.3 Analysis

Data were analysed for 21 clients who stayed at least one night in the South Australian MAP. Client summaries (e.g., investigations, medical conditions, medications, referrals) are described descriptively. Case notes (e.g., observational records) were imported into NVivo 1.7 (QSR International) and subject to inductive coding [22]. The coding framework was discussed and refined with the AGG. Ethics approvals were received from the Aboriginal Health Research Ethics Committee (Protocol # 04‐23‐1099) and the Southern Adelaide Clinical Human Research Ethics Committee (Protocol # 2023/HRE00340).

## 3 Results

### 3.1 Client Characteristics

Most clients identified as Aboriginal (*n* = 19) with a median age of 41 years. No clients identified as Torres Strait Islander. Five clients were managing 10 or more health conditions (Table 1).

**TABLE 1:** Client characteristics (*n* = 21).

Client characteristics	Total (*N* = 21)
Identity	
Aboriginal	19
Identified as a First Nations person from another colonised country	2
Age	
18–24 years	0
25–34 years	3
35–44 years	12
45–54 years	5
55+ years	1
Age summary statistics	
Mean (SD)	42 (7)
Median (IQR)	41 (37–49)
Min‐Max	28–56

Number of health conditions	
1 to 3 conditions	5
4 to 6 conditions	7
7 to 9 conditions	4
10 or more conditions	5
Health condition summary statistics	
Mean (SD)	7 (5)
Median (IQR)	6 (4–9)
Min‐Max	1–24

Abbreviation: IQR, interquartile range.

### 3.2 Program Engagement and Alcohol Consumption

The median duration of stay was 15 nights per client (range 1–48). As six clients entered the MAP twice (Table S1, Supporting Information), the median stay per admission was 14 nights (range 1–34) and the median time between re‐admissions was 17 nights (Table S2, Supporting Information). Six clients received a modified version of the MAP protocol.

Prior to entry, drinking patterns were characterised by: heavy episodic drinking – often from early morning to night; limited periods of abstinence; alcohol use in public spaces—often without food or shelter; chaotic drinking cycles marked by periods of extreme intoxication, withdrawal and repeat use without medical or psychosocial support; and high levels of involvement with emergency services (hospital presentations or police custody)—often directly related to alcohol use.

While alcohol cessation was not the aim of the MAP, six clients stopped drinking alcohol—four by withdrawing completely and two via ‘self‐weaning’ through gradual reduction under monitoring. Factors influencing the decision to modify consumption included personal or family reflection, noticing improvements, wanting to ‘give their body a break’ or improve health generally, or prompted by acute health issues (e.g., infection, injury, or underlying chronic illness) requiring monitoring and temporary abstinence.

### 3.3 Short Term Benefits

Short term benefits were categorised into five interrelated areas:

#### 3.3.1 Culture

Cultural support was provided by DASSA's Aboriginal Connection Program, a dedicated alcohol and other drug treatment service for Aboriginal peoples living within or frequenting the inner‐city area of Adelaide. Three client‐facing staff were Indigenous, and the cultural navigators identified with Kaurna^2^ and Ngarrindjeri^3^ communities. Pitjantjatjara language speakers were engaged from the Department of Human Services Remote Visitor Outreach Team that provides support services to remote visitors, particularly Aboriginal peoples, in Adelaide^4^. Culturally relevant diversionary activities included painting and art, burning bark, beading and weaving, and spending time outdoors to connect to Country. Kangaroo tail (*marlu wipu*) was cooked every second day, and clients were involved in cooking and teaching others. Clients were connected with Aboriginal‐led and Aboriginal Community Controlled Health Organisations. Some were transported to obtain *Mingkulpa* (a mixture of leaves and wood ash traditionally chewed as a stimulant). Three were supported to return to Country to participate in Sorry Business—the cultural obligations surrounding funerals.

#### 3.3.2 Medical Support

The MAP facilitated medical support in the form of accessible (e.g., coagulation studies, sexually transmitted infection assessment, infectious disease investigations and respiratory and function tests) and specialist (e.g., radiology, liver elastography, tissue sampling and endoscopy) health investigations (Table S3, Supporting Information). They were also provided with new/renewed prescription medication (Table S4, Supporting Information). Over half (*n* = 11) received at least one referral to a healthcare provider for a medical concern and almost one‐quarter (*n* = 5) received three referrals. The most referrals per client was four (*n* = 1). In addition, one client received two new medical diagnoses and five clients received one.

#### 3.3.3 Housing

The MAP provided a total of 399 nights of overnight accommodation. Clients were connected with mainstream housing services and two Aboriginal‐led housing organisations. Twelve clients were discharged to home/family and three secured housing.

#### 3.3.4 Government System Navigation

Clients were supported to navigate nine different types of government systems relating to income and other payments, the criminal justice system, legal support, health/disability insurance, child protection, human services and aged care. Outcomes included securing concession cards necessary for (subsidised) medical appointments and aged care packages, attending child access visits and receiving fines reductions.

#### 3.3.5 Capacity, Resilience and Social Connectedness

Clients were provided with information about on‐going alcohol harm reduction strategies, education and employment services. Some were transported to a local indigenous art gallery where they could sell their art. One client enrolled in a numeracy and literacy class at an Aboriginal Community College. Clients also received support with Activities of Daily Living [23] especially personal presentation and grooming, ambulating and feeding. They were also assisted to purchase communication devices necessary to connect with other people and the government systems outlined above.

## 4 Discussion

The South Australian MAP provided a culturally safe, holistic and responsive environment for developing less risky alcohol consumption patterns. Short term benefits addressed some barriers to healthcare commonly faced by people experiencing homelessness, such as a lack of transport, not being contactable and service costs [24], as well as difficulties navigating government systems [3]. Connections with formal institutions are particularly important, ‘as both homelessness and Indigeneity can be characterised by a distrust of formal institutions’ [3], p. 3. While MAPs cannot be expected to address broader systemic determinants of (ill) health [2, 25] this study suggests that they can be culturally tailored for Aboriginal peoples. However, benefits must be understood within a broader context of the long‐term chronic health risks associated with problematic alcohol consumption [26] and the need for on‐going support to secure/maintain housing as well as the skills and connections required for employment opportunities. Nonetheless, the success of this South Australian trial is pertinent, given the pressing need for more dedicated services for Indigenous Australians experiencing homelessness in urban areas [3].

### 4.1 Limitations and Further Research

Short‐term outcomes may not be maintained over the medium or longer term. Data linkage could enable the identification of benefits related to reduced interactions with other services, such as emergency departments and hospitals [27, 28, 29, 30]. Future programs could be tailored for people experiencing alcohol dependence amongst other Australian populations at risk of homelessness, such as people transitioning from health or social care arrangements, with experience of family and domestic violence, young people, children on care and protection orders, and older Australians [31].

## 5 Conclusion

People with harmful alcohol use experiencing homelessness face multiple barriers to accessing healthcare and community support, especially those additionally bearing systemic discrimination following colonisation, such as Aboriginal and Torres Strait Islander peoples. This article describes the short‐term impacts of the first MAP carried out in South Australia outside of COVID‐19 restrictions, and the first in Australia to be conducted in a healthcare setting. This evaluation supports international literature on MAPs as an effective harm reduction approach and strengthens evidence for the feasibility, acceptability, and benefits of MAPs in Australia.

## Author Contributions

Conceptualisation (Kirrilly Thompson, Jacqueline Bowden, Blaire Brewerton, Mark Thompson, Victoria Cock, Courtney Ryder), data curation (Kirrilly Thompson, Gianluca Di Censo, Blaire Brewerton, Mark Thompson, Victoria Cock), formal analysis (Kirrilly Thompson, Courtney Ryder, Gianluca Di Censo, AGG), funding acquisition (Jacqueline Bowden, Kirrilly Thompson, Courtney Ryder), research and investigation (all authors, AGG), methodology (all authors, AGG), supervision (Jacqueline Bowden, Victoria Cock, Kirrilly Thompson), visualisation (Kirrilly Thompson, Courtney Ryder, Gianluca Di Censo), writing – original draft preparation (Kirrilly Thompson, Jacqueline Bowden, Gianluca Di Censo, Neophytos Georgiou, Courtney Ryder), writing – review and editing (all authors, AGG). All authors have approved the submitted version.

## Conflicts of Interest

The authors report there are no competing interests to declare. Mark Thompson, Victoria Cock and Blaire Brewerton work for Drug and Alcohol Services South Australia. Kirrilly Thompson, Gianluca Di Censo, Jacqueline Bowden and Neophytos Georgiou received funding from the Australian Department of Health, Disability and Ageing to support research regarding alcohol and other drugs.

## Supporting information


**Table S1.** Duration of stay (nights) and number of admissions per client (*n* = 21).
**Table S2.** Duration of stay (nights) per MAP admission (*n* = 27).
**Table S3.** Number of health investigations per client (*n* = 21).
**Table S4.** Prescribed medications issued at discharge, per client (*n* = 21).

## Data Availability

Research data are not shared.

## Endnotes

^1^
A cultural navigator is an Aboriginal or Torres Strait Islander health professional who provides direct clinical care and cultural leadership within alcohol and other drug and broader health services. They support clients through culturally safe clinical care (e.g., assessments, home visits, referrals and health education). In addition to working directly with clients, they advise on culturally appropriate practices, contribute to staff training and education, and play a vital role in improving service design and delivery to build trust, increase access and improve health outcomes for Aboriginal peoples by bridging clinical care with cultural understanding.

^2^
Kaurna country refers to the Adelaide plains.

^3^
Ngarrindjeri country refers to the Lower Murray, Coorong and Lakes areas of South Australia.

^4^
Pitjantjatjara country refers mostly to the far north‐west of South Australia, extending into the Northern Territory and Western Australia, also known as the Aṉangu Pitjantjatjara Yankunytjatjara Lands.
