# Supplementary material for: Evaluation of a Residential Managed Alcohol Program for Aboriginal Peoples Experiencing Homelessness and Alcohol Dependence: Short‐Term Impacts of an Australian Trial
Source: Drug Alcohol Rev. 2025 Sep 2;44(7):2061–5. doi: 10.1111/dar.70025 (PMC12581936; doi:10.1111/dar.70025)
Supplement: Supplementary file 1 — Table S1. Duration of stay (nights) and number of admissions per client (n = 21). Table S2. Duration of stay (nights) per MAP admission (n = 27). Table S3. Number of health investigations per client (n = 21). Table S4. Prescribed medications issued at discharge, per client (n = 21). [file DAR-44-2061-s001.docx]

## Supporting Information

Table S1: Duration of stay (nights) and number of admissions per client (n=21)

| **Duration of stay (nights) and number of admissions** | **Total (N=21)** |
| --- | --- |
| Total nights in program | |
| 1-7 nights (one week) | 7 |
| 8-14 nights (two weeks) | 3 |
| 15-21 nights (three weeks) | 2 |
| 22-28 nights (four weeks) | 3 |
| 29+ nights (more than one month) | 6 |
| Summary statistics for duration of stay | |
| Mean (SD) | 19 (15) |
| Median (IQR) | 15 (6-32) |
| Min-Max | 1-48 |
| Number of MAP admissions | |
| 1 | 15 |
| 2 | 6 |
| Summary statistics for delay between readmissions | |
| Mean (SD) | 22 (18) |
| Median (IQR) | 17 (8-32) |
| Min-Max | 6-50 |

IQR, interquartile range; MAP, managed alcohol program.

Table S2: Duration of stay (nights) per MAP admission (n=27)

| **Duration of stay (nights) per MAP admission** | **Total (N=27)** |
| --- | --- |
| Consecutive nights in program | |
| 1-7 nights (one week) | 9 |
| 8-14 nights (two weeks) | 7 |
| 15-21 nights (three weeks) | 5 |
| 22+ nights (more than three weeks) | 6 |
| Summary statistics | |
| Mean (SD) | 15 (10) |
| Median (IQR) | 14 (7-22) |
| Min-Max | 1-34 |

IQR, interquartile range; MAP, managed alcohol program.

Table S3: Number of health investigations per client (n=21)

|  | **Total (N=21)** |
| --- | --- |
| Number of accessible assessments |  |
| 0 | 0 |
| 1-4 | 2 |
| 5-9 | 3 |
| 10-14 | 6 |
| 15+ | 10 |
| Summary statistics | |
| Mean (SD) | 13 (5) |
| Median (IQR) | 14 (10-16) |
| Min-Max | 3-22 |
| Number of specialist assessments | **Total (N=21)** |
| 0 | 12 |
| 1-3 | 4 |
| 4-6 | 5 |
| Summary statistics | |
| Mean (SD) | 2 (2) |
| Median (IQR) | 0 (0-3) |
| Min-Max | 0-6 |

IQR, interquartile range.

Table S4: Prescribed medications issued at discharge, per client (n=21)

|  | **Total (N=21)** |
| --- | --- |
| Number of different prescribed medications |  |
| 0 prescriptions | 2 |
| 1-3 prescriptions | 14 |
| 4-6 prescriptions | 5 |
| Summary statistics | |
| Mean (SD) | 2 (2) |
| Median (IQR) | 2 (1-3) |
| Min-Max | 0-6 |
| Restarted previously prescribed medications | |
| No | 12 |
| Yes | 9 |

IQR, interquartile range.
